# Wood in office spaces: The impact of different wooden furniture on aesthetic evaluation

**DOI:** 10.3389/fpsyg.2022.986627

**Published:** 2023-01-05

**Authors:** Yiwei Zhu, Qiang Wang, Feng Zhao

**Affiliations:** ^1^School of Design, Jiangnan University, Wuxi, China; ^2^College of Education, Psychology and Social Work, Flinders University, Adelaide, SA, Australia

**Keywords:** office space, wood color, wood coverage, aesthetic evaluation, wooden furniture

## Abstract

In modern urban life, individuals are spending an increasing amount of time in the office. However, working in an uncomfortable office space for extended periods can affect the physical and mental health of employees. On this basis, it is particularly important for employees to build a comfortable and healthy office environment that is conducive to their work. The present study aimed to explore the use of wood in office furniture to build a comfortable and healthy work environment. The use of wood in office spaces can effectively relieve the mental fatigue of employees. Focusing on wooden office furniture, this study explores its influence on the aesthetic evaluation of wooden office spaces by manipulating the wood color and coverage of the wooden furniture placed in office spaces. Experimenting with these changes will optimize the application of wood in office spaces, improve employees’ mental health. The results show that wood color and coverage significantly impact the aesthetic evaluation of wooden office spaces. People exhibit higher aesthetic evaluations of light and medium wood-colored office spaces and prefer spaces with low wood coverage. The findings of this study provide a reference for the use of wooden furniture to optimize workplaces.

## 1. Introduction

Due to technological and economic development, most people now work in offices (Lindberg et al., 2018). According the data from International Labor Organization (ILO), 2014 to 2020, Chinese workers worked an average of 42.95–47.77 h per week (Anttila et al., 2021), and 59% of Chinese employees worked in an office (Gensler, 2021). Working in an uncomfortable office space for extended periods would cause fatigue, negative emotions, and burnout (Johnson and Lipscomb, 2006; WHO, 2017; Haapakangas et al., 2018), and all of which impact workers’ mental health. Employee health issues are a prevalent and pressing issue worldwide, with data suggesting that the major global economies lose 4–6% of their GDP annually due to work-related health problems (WHO, 2019). Therefore, it is important to improve office environments to protect workers’ mental health.

As a space in which employees work inside for a long time, the physical characteristics of office spaces significantly impact employees’ mental health (Clements-Croome, 2006; Zhang et al., 2017; CDC, 2018). Specifically, the satisfaction of the office furniture (such as color, texture, and comfort) will affect the employees’ evaluation of the office environment (Kim and De Dear, 2012), while an office environment with a positive aesthetic evaluation is beneficial to the mental health of employees (Schell et al., 2011; Li and Yu, 2020). Therefore, there is a pressing need to optimize furniture design in office spaces to improve employees’ aesthetic evaluation with office environment and consequently improve their mental health. To this end, this study aimed at investigating the effect of different furniture designs (e.g., designs with different wood colors and wood coverage) on employees’ aesthetic evaluation with their office environment.

Environmental psychology research showed that built environments with natural elements were more popular and more positively evaluated by people (Ulrich, 1983; Brandt and Shook, 2005). Attention Recovery Theory (Kaplan and Kaplan, 1989) and Psychological Recovery Theory (also known as Stress Reduction Theory, Ulrich, 1991) posit that introducing natural elements into the built environment can effectively help people recover from psychological depletion and improve their psychological wellbeing more generally (Burnard et al., 2017; Li et al., 2022). Specifically, Attention Recovery Theory states that natural views provide stimulation which activates undirected attention and restores depleted attention system (Kaplan and Kaplan, 1989; Kaplan, 1995), while Stress Reduction Theory believed that natural environments could aid recovery from stressful events, block negative thoughts, and turn emotions to positive side, help people restore healthy cognition and behavior (Ulrich, 1983; Ulrich, 1991; Ulrich et al., 1991). So, from the perspective of Stress Reduction Theory, it was reasonable to argue that the natural environment can promote positive emotions and improve mental health. Some studies had examined bringing nature into the indoor environment. For example, Kahn et al. (2008) found that working in the office with a glass window on natural views had a better recovery of psychological stress compared to that with blank wall. Yin et al. (2018) found that participants’ negative emotions decreased and positive emotions increased after they had used an office space with natural elements (such as natural lighting and plants). These suggest that the restorative environment built by introducing natural elements can help people recovery from psychological stress and improve their mental health. Therefore, the use of natural elements in the office space to create a restorative office environment where employees are satisfied is beneficial to their mental health.

There are many ways in which natural elements can be incorporated into buildings (e.g., views, water features, plants, natural materials, variations in shape, lighting, etc.). The introduction of natural material remains a simple and widely accessible means to bring nature elements to indoors environment (Kellert, 2008; Burnard et al., 2017). Wood is a widely used natural material that is often used in building structures, furniture, and decorative items due to its easy acceptance for consumers and easy processing properties (Noora et al., 2020). It has been found that wood has significant positive effects on people’s health, such as improving concentration and comfort and enhancing sense of security (Sakuragawa et al., 2008; Grote et al., 2009; Fell, 2010; Alapieti et al., 2020; Lipovac and Burnard, 2021). Furniture is an essential and practical appliance in office spaces, and people has a positive attitude toward wooden office furniture (Ridoutt et al., 2002; Paluš et al., 2012). Therefore, this study took wooden furniture as an entry point to improve the aesthetic evaluation of employees in office spaces. Through the thoughtful use of wooden furniture to create a healthy and relaxing office environment for employees.

Aesthetic evaluation pertains to the participant’s assessment of an object’s aesthetic value; it is the embodiment of people’s aesthetic attitude toward things (Zhu, 2012). Aesthetic evaluation is an essential reference point in the design optimization of wooden furniture, and an important indicator of design merit in product, spatial, and architectural design. Aesthetic evaluation is relatively complex, involving aspects of visual perception, affective responses, tendency to act, etc. (Ajzen, 2005). Specifically, visual perception is a person’s sensory evaluation of the beauty of things derived from their content and form. Affective response is people’s preference or satisfaction degree toward an object (Nyrud et al., 2010). Whilst, the tendency to act refers to an individual’s willingness to use things.

People’s evaluations of wood environments are closely related to the physical properties of wood, such as tree species, color, knot count, cover area, etc. (Nakamura and Kondo, 2008; Høibø and Nyrud, 2010; Fujisaki et al., 2015; Manuel et al., 2015). It should be noted that people exhibit different attitudes toward different types of wood. For example, oak is considered masculine, whilst mahogany is considered feminine (Blomgren, 1965). Tang (2021) divided the wood color into light wood colors (represented by oak and pine), medium wood colors (represented by basswood and teak), and dark wood colors (represented by hickory and black walnut) according to the material’s brightness. Study has demonstrated that wood color has a notable impact on the overall effect of the wood environment. For example, Burnard et al. (2017) found that people using lighter colored oak office environment generated lower levels of stress compared to the darker colored walnut office environment, when all other physical conditions were the same. Therefore, different wood colors (light wood-colored, medium wood-colored, and dark wood-colored) were counted as an independent variable in the present study, to investigate its role in the aesthetic evaluation of wooden office spaces. Based on the study of Burnard et al. (2017), this study hypothesized that lighter colored wooden office spaces have a more positive aesthetic evaluation.

Wood cover, another important variable affecting the evaluation of woody environments, is the ratio of wood surface area to space surface area (Rice et al., 2006; Nyrud et al., 2010; Lipovac et al., 2020). Elsewhere, a direct correlation between woody environmental assessment and wood cover has been identified (Masuda, 1988; Masuda and Nakamura, 1990; Nyrud et al., 2010). Tsunetsugu et al. (2007) found that the room with 45% wood coverage was the most favored among the three rooms (0, 45, and 90%), and the 90% covered room was rated as the most uncomfortable. Nyrud et al. (2014) found that hospital employees prefer the mediate levels of wood decoration in patient rooms (wood on the walls, floor), followed by traditional patient rooms without the inclusion of wood, and all-wood patient rooms (wood on all the walls, floor, ceiling, and furniture) were the least popular patient rooms. It seems lower coverage wood spaces were evaluated more positively than the higher coverage wood spaces. Therefore, this study hypothesized that the aesthetic evaluation of wooden office spaces with lower-coverage is more positive.

In summary, this study examined the aesthetic evaluation of wooden office spaces when different types of wood are used. It aimed to improve the mental health of employees by building the satisfying wooden office space through the reasonable use of wood in office furniture. However, in current furniture production, various wood-based composites are used to replace solid wood, such as plywood, particle board, MDF, etc. (Burnard and Kutnar, 2015). On this basis, the present study did not consider the wood species and number of knots in the wood environmental assessment. In addition, because wood knots are, to a degree, easily affected by wood color and deepening wood color can cover the defects of excessive wood knots on the surface. As such, wood knots were not considered in this article. To be precise, this study examined the effects of wood color and wood cover on the aesthetic evaluation of wooden office spaces. As noted above, the aesthetic evaluation involves three aspects of visual perception (people’s sensory evaluation of the wooden office space), affective responses (degree of satisfaction) and tendency to act (usage willingness).

## 2. Materials and methods

### 2.1. Participants

This study recruited 185 adults aged 18–57 years (*M*_age_ = 28.37 ± 7.83 years, 78 men, 107 women) online to participate in this trial (Supplementary Data Sheet 1). Among them, the participants worked or studied indoors for an average of 7.77 ± 2.03 h per day, with an average of 6.21 ± 8.31 years of work experience. In the study sample, 30.30% of the participants had college degrees or below, 50.30% had bachelor’s degrees, 16.80% had master’s degrees, and 2.7% had doctoral degrees. All participants reported that they had normal vision and did not suffer from any no visual defects, such as color blindness. At the end of the experiment, each person was paid 10 RMB as compensation.

### 2.2. Experimental design

The experiment conducted in this research adopted a two-factor between-subject design of 3 (wood color: light #ECCD97, medium #ECB36C, dark #95582C) × 2 (wood coverage: low 12%, high 35%), with wood color and wood coverage as the independent variables. Meanwhile, sensory evaluation, satisfaction, and usage willingness of office space make up the dependent variables. In total, six experimental conditions levels were formed: light wood-colored and low-coverage wood (light and low), light wood-colored and high-coverage wood (light and high), medium wood-colored and low-coverage wood (medium and low), medium wood-colored and high-coverage wood (medium and high), dark wood-colored and low-coverage wood (dark and low), and dark wood-colored and high-coverage wood (dark and high).

Three hypotheses were consequently put forth in this study:

**H1:** Wood color and wood coverage affect people’s sensory evaluation of wooden office space, with people evaluating light wood-colored and low-coverage wooden office spaces more positively.

**H2:** Wood color and wood coverage affect people’s satisfaction with wooden office spaces, with people being more satisfied with light wood-colored and low-coverage wooden office spaces.

**H3:** Wood color and wood coverage affect people’s usage willingness of wooden office spaces, with people more willing to use light wood-colored and low-coverage wooden office spaces.

### 2.3. Materials

#### 2.3.1. Video production

This study aims to examine people’s sensory evaluation, satisfaction, and willingness to use different wooden spaces; as such, the setting of wooden offices is the crux of this experiment. In this experiment, Lumion8.0 software was used to produce six videos with a duration of 35 s and a resolution of 720 dpi to show the six kinds of wooden office spaces mentioned above (Supplementary Videos 1–6). In these six types of wooden spaces, except for wooden furniture, the interior construction (including ceiling, walls, floor, windows, etc.), lighting settings (including direction, heating and cooling, intensity, etc.), and the arrangement of other objects remained consistent.

**FIGURE 1:**
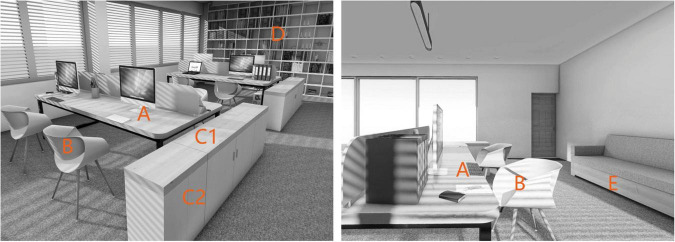
Wooden office space wood application location diagram. Reproduced with permission from https://www.6mo.cn.

The wooden spaces were each furnished with two desks, eight chairs, two storage cabinets, one floor-to-ceiling bookcase, and one sofa. Additionally, soft lighting with natural light illumination was used to highlight the appearance of typical natural office spaces. As shown in Figure 1 and Table 1, wood was applied in six areas: desk top (A), office chair seat surface (B), counter top and cabinet body of storage cabinet (C1 and C2), bookcase (D), and sofa stents (E). In the high-coverage condition, all these areas were covered with wood, total 35% wood coverage for the overall office. In the low-coverage condition, only area A, C1, and E were covered with wood, other areas were used other material, specifically, B used beige (#E8D1A8) plastic material, C2 and D used beige (#E8D1A8) metal material, total 12% wood coverage for the overall office. It should be noted that the changes in wood color were also presented on these six areas. In total, six kinds of wooden spaces were formed: light and low, light and high, medium and low, medium and high, dark and low, and dark and high (Table 1). The final appearance of the six different experimental conditions is shown in Table 2.

**TABLE 1:** Wood colors and wood areas in office space.

	Desk top (A)	Office chair seat surface (B)	Counter top of storage cabinet (C1)	Cabinet body of storage cabinet (C2)	Bookcase (D)	Sofa stents (E)
Light and low	Light color wood	Plastic material	Light color wood	Metal material	Metal material	Light color wood
Light and high	Light color wood	Light color wood	Light color wood	Light color wood	Light color wood	Light color wood
Medium and low	Medium color wood	Plastic material	Medium color wood	Metal material	Metal material	Medium color wood
Medium and high	Medium color wood	Medium color wood	Medium color wood	Medium color wood	Medium color wood	Medium color wood
Dark and low	Dark color wood	Plastic material	Dark color wood	Metal material	Metal material	Dark color wood
Dark and high	Dark color wood	Dark color wood	Dark color wood	Dark color wood	Dark color wood	Dark color wood

**TABLE 2:** The six different experimental condition.

	Light wood-colored	Medium wood-colored	Dark wood-colored
Low-cover wood	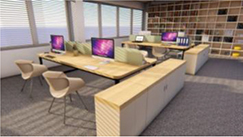	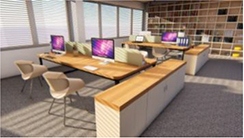	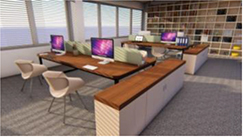
High-cover wood	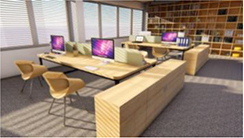	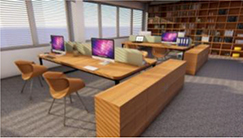	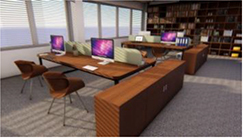

Reproduced with permission from https://www.6mo.cn.

#### 2.3.2. Picture creation

To assist the study participants to imagine themselves working in a wooden office space, this experiment on the basis of the video to construct different scenes of the office spaces. Four scenes were produced for each wooden office space (Supplementary Image 1). A total of 24 scene pictures with resolutions of 300 dpi were produced for the six wooden office spaces using Photoshop 19.0 software (Supplementary Image 1, Supplementary Figures 7–11). Supplementary Figures 7–11, Supplementary Videos 1–6, Supplementary Data Sheet 1, and Supplementary Image 1 were placed in the Supplementary material.

### 2.4. Measurements

#### 2.4.1. Positive affect negative affect scale

This study measured the participant’s state of emotional before the formal experiment in order to control the influence of emotion on the participant’s sensory evaluation, satisfaction, and usage willingness. The scale of emotion was derived from the Positive Affect Negative Affect Scale (PANAS), as by revised Qiu et al. (2008). This scale is divided into two dimensions, positive emotion and negative emotion, and consists of 18 adjectives. For example, positive emotions (Cronbach’s α = 0.93) include “active,” “enthusiastic,” and “happy” et al. (“*at the present moment, I feel I am…* “), whilst negative emotions (Cronbach’s α = 0.93) include “shame,” “sadness,” and “fear” et al. (“*at the present moment, I feel I am…* “). Items were scored on a 7-point scale, with higher scores indicating stronger positive or negative emotions.

#### 2.4.2. Sensory evaluation scale

The sensory evaluation scale for wooden office spaces was adapted from scales utilized by Rice et al. (2006) and Yildirim et al. (2011) in their respective research. The scale in the present study consists of 10 adjectives: “bright,” “spacious,” “organized,” “relaxing,” “natural,” “harmonious,” “vivid,” “comfortable,” “beautiful,” and “attractive,” to match the question “*what do you think of the office environment?*” The Chinese version of the scale was revised by bilingual Chinese-American and corrected by professional translators. The scale is scored on a 7-point scale, with higher scores indicating better sensory evaluations. Moreover, the internal consistency reliability of the sensory evaluation scale was α = 0.93.

#### 2.4.3. The satisfaction and usage willingness relating to wooden office spaces

Satisfaction with wooden office furniture in office space of this study is measured by administering two questions: “How satisfied are you with your office environment?” and “If you worked here, to what extent does this wooden furniture meet your demands for an office environment?” A 7-point scale was used to measure satisfaction, with higher scores indicating a greater degree of satisfaction. The Cronbach’s α of these two items was 0.88. Meanwhile, the usage willingness an individual feels toward a wooden office space was also measured by two items: “How much do you like to work here?” and “How willing are you to work here?” A 7-point score was also used, and again, the higher the score, the greater the willingness to use the wooden office space. The Cronbach’s α of these two items was 0.87.

### 2.5. Procedure

We recruited participants online, the questionnaire.com platform was used to present our research materials and measurements. Participants scanned the QR code or logged into the URL to participate in the present experiment. They were randomly assigned to one of six different experimental conditions. After carefully reviewing the study instructions, the participants first took a mood measurement (PANAS scale). They then watched a 35-s video of a wooden office space. After watching the video, the participants were asked to perform an imaginary task, that is, spend 1 min imagining themselves working and communicating in the office environment presented in the video. To assist with this, four drawings of the wooden office space were presented showing the scene from different angles. After completing the imagination task, the participants were asked to evaluate the office space in terms of sensory evaluation, satisfaction, and willingness to use. Finally, the participants filled in their demographic information, which marked the end of the experiment.

### 2.6. Data statistics and analysis

After examining the normality of the data, two-way ANOVA and non-parametric tests (Mann–Whitney test and Kruskal–Wallis test) were used as the methodology for statistical analysis. Wood color and wood coverage were taken as the independent variables, whilst sensory evaluation, satisfaction, and usage willingness were taken as the dependent variables. Positive and negative emotional states were used as covariates. Statistical analysis was performed using SPSS 20.0. To verify hypothesis 1, 2, and 3, this study examined the main effects of wood color and wood cover on the participants’ sensory evaluation, satisfaction, and usage willingness.

## 3. Results

### 3.1. Aesthetic evaluation of the six different experimental conditions

**FIGURE 2:**
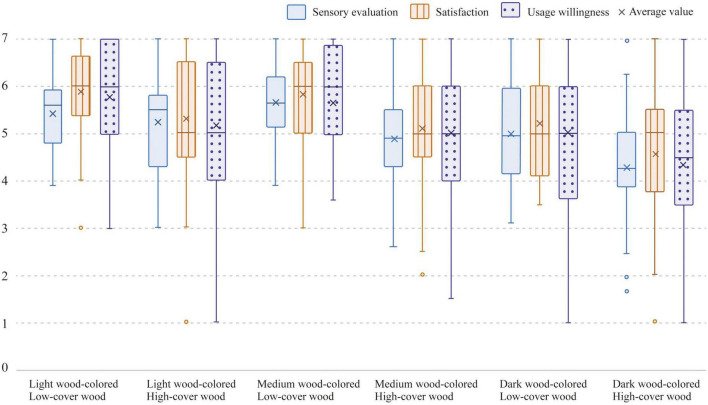
Aesthetic evaluation scores for the six different experimental conditions.

Figure 2 details the aesthetic evaluation scores for six different experimental conditions. Specifically, aesthetic evaluation includes three aspects: sensory evaluation, satisfaction, and usage willingness. The results showed that medium wood-colored and low-coverage wooden offices were the most highly rated in terms of sensory evaluation, whilst light wood-colored and low-coverage wooden offices received the highest scores in terms of satisfaction and usage willingness. By way of contrast, dark wood-colored and high-cover wooden offices received the lowest evaluations in the three aspects noted above.

### 3.2. The descriptive statistics and correlations among the variables

The correlation analysis was used to investigate the correlations among dependent variables, the results showed that there was a significantly positive correlation among sensory evaluation, satisfaction, and usage willingness, Table 3 details the correlations among variables.

**TABLE 3:** The descriptive statistics and correlations between the variables.

	Sensory evaluation	Satisfaction	Usage willingness
Sensory evaluation	1		
Satisfaction	0.81**	1	
Usage willingness	0.82**	0.87**	1
*M*	5.11	5.35	5.16
SD	1.06	1.30	1.46

**Correlation is significant at the 0.01 level (two-tailed).

### 3.3. Effects of wood color and wood coverage on sensory evaluation

Because null hypothesis was retained (*p* = 0.20 > 0.05) in the normal distribution test, which represents the distribution of sensory evaluation is normal, two-way ANOVA was chose as the statistical analysis method. The results passed Levene’s test of equality of variances, *F*(5,179) = 0.76, *p* = 0.576 and showed a significant main effect of wood color, *F*(2,177) = 6.95, *p* = 0.001, $\eta_{p}^{2}=0.07$, indicating that wood color had a significant impact on sensory evaluation. *Post-hoc* tests (LSD) of wood color revealed significant differences in the visual perception of dark wood-colored (*M* = 4.65 ± 1.24) and light wood-colored (*M* = 5.34 ± 0.89) offices, and also between dark wood-colored and medium wood-colored (*M* = 5.28 ± 0.93) offices, *p* = 0.002, *p* = 0.001. Contrastingly, no significant difference was found between light wood-colored and medium wood-colored wood, *p* = 0.889. The main effect of wood coverage was significant, with the visual perception of the low-coverage wooden space (*M* = 5.38 ± 0.96) being significantly better than that of the high-coverage wooden space (*M* = 4.83 ± 1.10), *F*(1,177) = 7.22, *p* = 0.008, $\eta_{p}^{2}=0.04$. However, the interaction between wood color and wood coverage was not significant, *F*(2,177) = 1.72, *p* = 0.182. Table 4 presents the sensory evaluation scores under different experimental conditions.

**TABLE 4:** Sensory evaluation score.

Wood color	Wood coverage	*M* ± SD	*N*
Light wood-colored	Low-coverage	5.43 ± 0.80	34
	High-coverage	5.24 ± 1.00	31
	Total	5.34 ± 0.89	65
Medium wood-colored	Low-coverage	5.70 ± 0.79	32
	High-coverage	4.97 ± 0.90	31
	Total	5.34 ± 0.92	63
Dark wood-colored	Low-coverage	5.04 ± 1.19	28
	High-coverage	4.38 ± 1.19	29
	Total	4.71 ± 1.23	57
Total	Low-coverage	5.41 ± 0.96	94
	High-coverage	4.90 ± 1.08	91
	Total	5.16 ± 1.05	185

### 3.4. Effects of wood color and wood coverage on satisfaction

Because null hypothesis was rejected (*p* < 0.001) in the normal distribution test, which represents the distribution of satisfaction is not normal, Kruskal–Wallis test and Mann–Whitney test were chose as statistical analysis methods. The Kruskal–Wallis H test showed that there was a statistically significant difference in satisfaction score among the different wood-colored offices, χ^2^(2) = 11.95, *p* = 0.003, with a mean rank satisfaction of 105.12 for light wood-colored offices (*M* = 5.60 ± 1.27), 98.34 for medium wood-colored offices (*M* = 5.48 ± 1.21) and 73.27 for dark wood-colored offices (*M* = 4.88 ± 1.31). The Mann–Whitney U test indicated that satisfaction in the low-coverage wooden space (*M* = 5.66 ± 1.09) was statistically significantly higher than the high-coverage wooden space (*M* = 5.00 ± 1.41) (*U* = 3,095.00, *p* = 0.001). Table 5 details the satisfaction scores under different experimental conditions.

**TABLE 5:** Satisfaction score.

Wood color	Wood coverage	*M* ± SD	*N*
Light wood-colored	Low-coverage	5.87 ± 1.07	34
	High-coverage	5.31 ± 1.42	31
	Total	5.60 ± 1.27	65
Medium wood-colored	Low-coverage	5.83 ± 1.02	32
	High-coverage	5.11 ± 1.30	31
	Total	5.48 ± 1.21	63
Dark wood-colored	Low-coverage	5.21 ± 1.09	28
	High-coverage	4.55 ± 1.44	29
	Total	4.88 ± 1.31	57
Total	Low-coverage	5.66 ± 1.09	94
	High-coverage	5.00 ± 1.41	91
	Total	5.34 ± 1.30	185

### 3.5. Effects of wood color and wood coverage on usage willingness

Because null hypothesis was rejected (*p* < 0.001) in the normal distribution test, which represents the distribution of usage willingness is not normal either, Kruskal–Wallis test and Mann–Whitney test were chose as statistical analysis methods. The Kruskal–Wallis H test showed that there was a statistically significant difference in usage willingness score among the different wood-colored offices, χ^2^(2) = 12.93, *p* = 0.002, with a mean rank usage willingness of 105.29 for light wood-colored offices (*M* = 5.48 ± 1.42), 99.04 for medium wood-colored offices (*M* = 5.36 ± 1.32) and 72.31 for dark wood-colored offices (*M* = 4.59 ± 1.52). The Mann–Whitney U test indicated that usage willingness in the low-coverage wooden space (*M* = 5.47 ± 1.36) was statistically significantly higher than the high-coverage wooden space (*M* = 5.16 ± 1.46) (*U* = 3250.00, *p* = 0.004). Table 6 lists the scores of usage willingness under different experimental conditions.

**TABLE 6:** Usage willingness score.

Wood color	Wood coverage	*M* ± SD	*N*
Light wood-colored	Low-coverage	5.78 ± 1.19	34
	High-coverage	5.16 ± 1.58	31
	Total	5.48 ± 1.42	65
Medium wood-colored	Low-coverage	5.69 ± 1.25	32
	High-coverage	5.02 ± 1.31	31
	Total	5.36 ± 1.32	63
Dark wood-colored	Low-coverage	4.83 ± 1.48	28
	High-coverage	4.34 ± 1.54	29
	Total	4.59 ± 1.52	57
Total	Low-coverage	5.47 ± 1.36	94
	High-coverage	4.85 ± 1.51	91
	Total	5.16 ± 1.46	185

## 4. Discussion

This study experimentally examined the research participants’ aesthetic evaluations of wooden office spaces featuring different wood colors and wood coverage. Based on the results, there were significant differences in wood color in terms of sensory evaluation, satisfaction, and usage willingness. Compared with dark wood office spaces, the study participants exhibited better sensory evaluations, higher satisfaction levels, and a stronger willingness to use light and medium wood-colored office spaces. In addition, there are significant differences in wood coverage rate in terms of sensory evaluation, satisfaction, and usage willingness: specifically, low-coverage wooden office spaces were found to be superior to the high-coverage wooden office spaces in terms of sensory evaluation, satisfaction, and usage willingness. From this, it can be concluded that light wood-colored furniture in low-coverage wooden office spaces is more aesthetically pleasing.

### 4.1. Wood color and wood coverage influence sensory evaluation

People exhibited a better, more positive sensory evaluation of light and medium wood-colored wooden office spaces than in dark wood-colored office spaces. The reason for this is most likely that light and medium wood-colored furniture with higher lightness creates wooden spaces that are bright, spacious, and relaxing. Contrastingly, dark wood-colored furniture with lower lightness creates wooden spaces that are dim, narrow, and tense. This is consistent with previous findings, namely that spaces created using light wood-colored oak are considered pleasant and comfortable, whereas spaces created with dark wood-colored black walnut are considered depressing and closed (Poirier et al., 2019). In addition, when given a choice of spatial color, the study participants tended to select brighter colors, regardless of hue (Hidayetoglu et al., 2012). The reason why low-coverage wooden spaces are evaluated more positively compared to high-coverage wooden office spaces may be that people perceive low-coverage wood spaces to be aesthetically pleasing and vivid, whilst too much wood coverage can be regarded as unduly constricting (Li et al., 2021).

As a natural material, the unique texture, color, and other surface characteristics of wood can allow people to perceive the beauty of nature, thus leading individuals in wooden spaces and environments to derive a comfortable, relaxed, and natural feeling (Rice et al., 2006). However, at the same time, the texture and color of wooden surfaces increase the complexity of space design. Therefore, moderate use of wood can enrich the design of a space and make it vivid and interesting. However, wood should be used thoughtfully, as excessive use tends to make the space appear narrow and complicated, resulting in a sense of depression and feelings of burden. This explains why the study participants preferred wooden office spaces with low-coverage over those with high-coverage. Previous studies have also found that spaces with wood coverage of around 45% also received the highest scores for comfort and relaxation (Masuda and Nakamura, 1990; Tsunetsugu et al., 2007).

### 4.2. Wood color and wood coverage influence satisfaction

Similarly, the participants were found to exhibit greater satisfaction with light and medium wood-colored office spaces compared to dark wood-colored office spaces. Notably, the participants were not only satisfied with the light (medium) wood-colored office spaces, but also with the light (medium) wood-colored furniture positioned in the space. This is consistent with previous studies that also verified this result, such as Scholz and Decker (2007), who observed that German consumers were most satisfied with light wood-colored oak furniture. In addition, light wood color oak furniture is not only more satisfying to consumers, but also helps to alleviate feelings of stress. For example, Burnard and Kutnar (2020) found that experiment participants produced lower concentrations of salivary cortisol in an oak office environment compared to a walnut office environment, when all other same physical conditions were the same. This indicates that oak office furniture can be used to generate lower levels of stress amongst office workers. However, it is worth noting that people are more satisfied with dark wood-colored wooden products in terms of product design (Wan et al., 2021); such a difference may stem from the fact that the general product is smaller in size and used alone. Although furniture falls within a category of products, it is bulky and often exists in the building space in combination with other products and fixtures. Compared with a single product, a complete set of furniture has a greater visual impact, thus leading individuals to be more satisfied with light and medium wood-colored furniture than dark wood-colored furniture. In terms of furniture coverage, people are more satisfied with low-coverage wood composite furniture than with high-coverage all-wood furniture. For example, Turkish consumers have been found to prefer wood composite furniture with partial use of wood to all-wood furniture. This is premised on their belief that all-wood furniture is expensive and wood composite furniture can reduce costs whilst also offering greater design possibilities (Guzel, 2020).

### 4.3. Wood color and wood coverage influence usage willingness

As indicated by the results, individuals demonstrated a stronger intention to use light (medium) wood-colored furniture and low-coverage wooden spaces, that is, they prefer to work in office spaces with light or medium wood-colored furniture and low-coverage wood. There are several possible reasons for this. Firstly, light and medium wood-colored conform to the aesthetic needs of employees. Many traditional Chinese-style residential environments use dark-colored woods to make residents feel stable and solemn. However, employees who are accustomed to a faster pace of life, heavy workloads, and excessive work pressure prefer to use relaxed and vivid natural light and medium wood-colored furniture in their office space. Secondly, the use of light and medium wood-colored can enhance the lighting of a space and create a warm atmosphere, thus reducing power consumption (Jafarian et al., 2018). This is an especially important consideration at high latitudes and in cold climates. Thirdly, because the dimensional instability of wood exposed to wet conditions limits long-term use of wood (He et al., 2020), low-coverage wood spaces are more stable compared to high-coverage wood spaces, whilst also reducing the use of wood and alleviating wood supply shortages to a certain extent. The latter consideration is especially important for countries with scarce forest resources, such as China.

### 4.4. Theoretical contributions and practical implication

This study has its advantages in the exploration of the application of wood spaces. First, this study examined wooden spaces by the dual dimensions: wood color and wood coverage. It made the present research more comprehensive than others. For example, in the study by Burnard and Kutnar (2020), researchers compared people’s stress responses to different wooden color furniture in office environments, and Song et al. (2016) examined people’s preferences in different wooden office environments by changing the wood coverage in office spaces. However, both studies examined people’s attitudes and responses to wood environments by just only controlling one physical characteristic of wood. Thus, neither study was not comprehensive enough. Second, this study set a specific wooden office space and identified its functional properties of the space, which made the present study more specific in the application of wood space. Li et al. (2021) explored the effects of different degrees of wooden use in interior spaces on people’s psychological responses and visual impressions. Similarly, Strobel et al. (2017) investigated the best way to use wood in interior spaces. Both studies did not clarify the functional properties of interior spaces, which means they were not clear enough to study wooden spaces.

Rapid economic development has exposed both companies and employees to intense competition and tremendous stress, which has made mental health problems in the workplace prevalent worldwide (Mackenzie, 2019; Prada-Ospina, 2019; Herr et al., 2022). It is important to note that a good working environment is a prerequisite for employees’ occupational health and occupational wellbeing (Danna and Griffin, 1999). While wood is an ideal material for restorative design, and the effective use of wood in interior environments is an important means of building restorative environments (Nyrud and Bringslimark, 2010; Derr and Kellert, 2013; Demattè et al., 2018). Moreover, many studies have demonstrated that the use of wood in architectural interiors generally produces positive effects on the physical and mental health of occupants, such as lowering heart rate and blood pressure, relieving psychological stress, and alleviating visual fatigue (Tsunetsugu et al., 2002; Fell, 2010; Zhang et al., 2016; Hirata et al., 2017). However, this does not guarantee that the use of one type of wood will improve health, nor does it mean that the more types of wood that are used, the better the results will be. Therefore, it is important to explore what kind of wood to use and how to use it.

This study examined ways to optimize the workplace *via* the sensible use of wood in office furniture, to enhance the satisfaction of workspaces and eventually benefit the mental health of employees. On the one hand, the results of this study provided guidance for the design of wooden office spaces and office furniture on a global scale. On the other hand, the findings provide scientific evidence for the rational and effective use of wood in restorative environment. Constructing restorative environments is helpful for recovery from psychological and physical stresses (Danna and Griffin, 1999), which may be the main reason for the increasing research on wood and its application in the fields of architecture, psychology, and design.

### 4.5. Limitations and future research

Although the results of this study have reference value for the design of wooden office spaces, it nevertheless is subject to certain shortcomings. First, there is a lack of consideration of the participant population and insufficient diversity within the study sample, thus limiting the generalizability of the study results. For example, there are differences in the needs of people with different working positions and different health conditions in relation to wooden office spaces. Second, the position of wood on wooden furniture may also affect people’s attitudes. For example, Kayseri consumers favor wooden desk tops (Guzel, 2020). However, the consideration of the application position of wood was neglected in this study, so there is a need to increase the consideration of the application position of wood in future studies of wooden spaces. Third, this study only involved the visual senses, and the participants may have a weaker sense of experience in the office context when exclusively examined in relation to the visual senses. Future research should focus on other forms of sensory evaluation, such as touch, smell, etc. Incorporating other sensory experiences may improve the problem of a weaker sense of experience and render the aesthetic evaluation of different wooden office spaces more comprehensive and objective. To help overcome the constraints of the environmental experience, in future studies, participants can wear immersive helmets or glasses to enable multi-person interaction, simulating real office scenarios. In addition, participants can also have the real touch feeling of wood by wearing tactile gloves. So as to enhance the experience of wooden office spaces. Last, to further explore people’s sensory evaluation, affective responses, and tendency to act toward wooden spaces, future studies may take into account including eye-movement experiments and EEG experiments.

## 5. Conclusion

This study investigated the effects of wood color and wood coverage on the aesthetic evaluation (sensory evaluation, satisfaction, and usage willingness) of wooden office spaces. From the results, it was found that people exhibit better aesthetic evaluations of light and medium wood-colored office spaces and prefer wood office spaces with low wood coverage.

These findings could help designers to better live up to employees’ expectations of wooden office spaces. Moreover, they can provide a useful reference for the rational use of wood, the optimization of office space design, and restorative environmental design. On the one hand, considering that China is a significant global consumer and producer of wood products, the present study might provide a valuable reference to local furniture manufacturers regarding the sensible use of wood in office furniture. On the other hand, this study also provided crucial information on enhancing employees’ mental health from an interior design perspective in light of the widespread issue of mental health in the workplace.

## Data availability statement

The original contributions presented in this study are included in the article/Supplementary material, further inquiries can be directed to the corresponding authors.

## Ethics statement

Ethical review and approval was not required for the study on human participants in accordance with the local legislation and institutional requirements. The patients/participants provided their written informed consent to participate in this study.

## Author contributions

YZ wrote the manuscript and involved in all steps of the research process. QW made a substantial, direct, and intellectual contribution to this work. FZ was responsible for experimental design, data analysis, and manuscript revision. All authors contributed to the article and approved the submitted version.

## Supplementary material

Supplementary Image 1 Schematic diagram of the use scene of wooden office furniture (medium and low).
