# Supplementary material for: Wood in office spaces: The impact of different wooden furniture on aesthetic evaluation
Source: Front Psychol. 2023 Jan 5;13:986627. doi: 10.3389/fpsyg.2022.986627 (PMC9849947; doi:10.3389/fpsyg.2022.986627)
Supplement: Supplementary file 1 [file Data_Sheet_1.zip › Supplementary_Material/Supplementary_Material.docx]

Supplementary Material

# Supplementary Data

Supplementary Data. Aesthetic evaluation scores for the six different experimental conditions

# Supplementary Figures and Tables

## Supplementary Figure

##
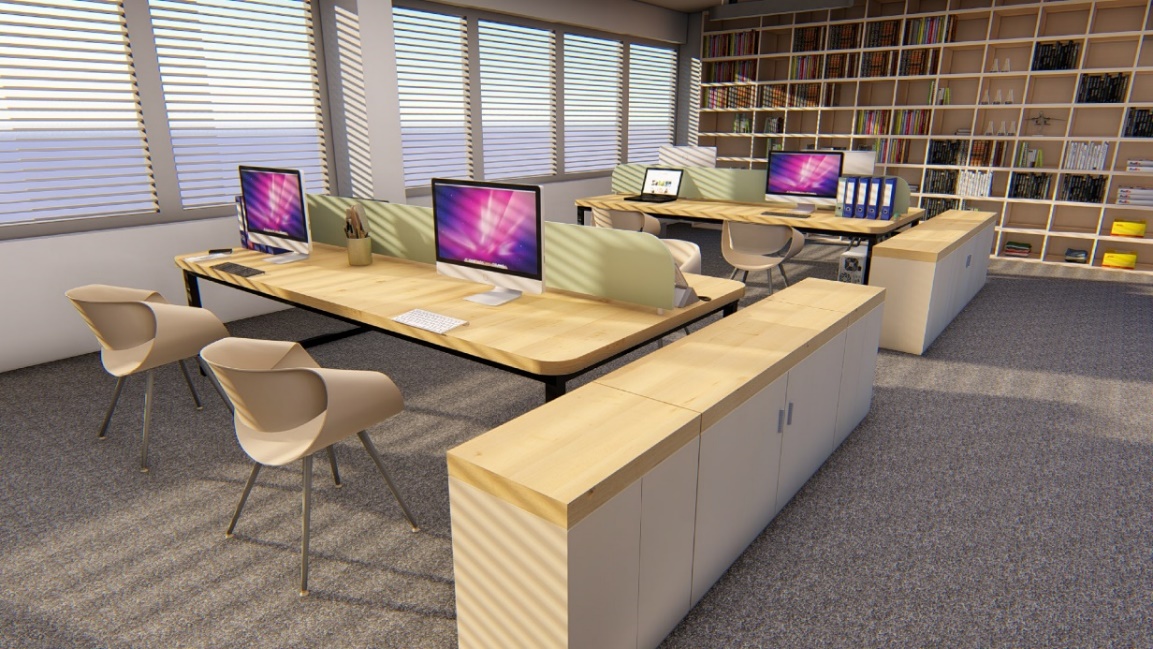


## Supplementary Figure 1. Light wood-colored and low-coverage wood

##
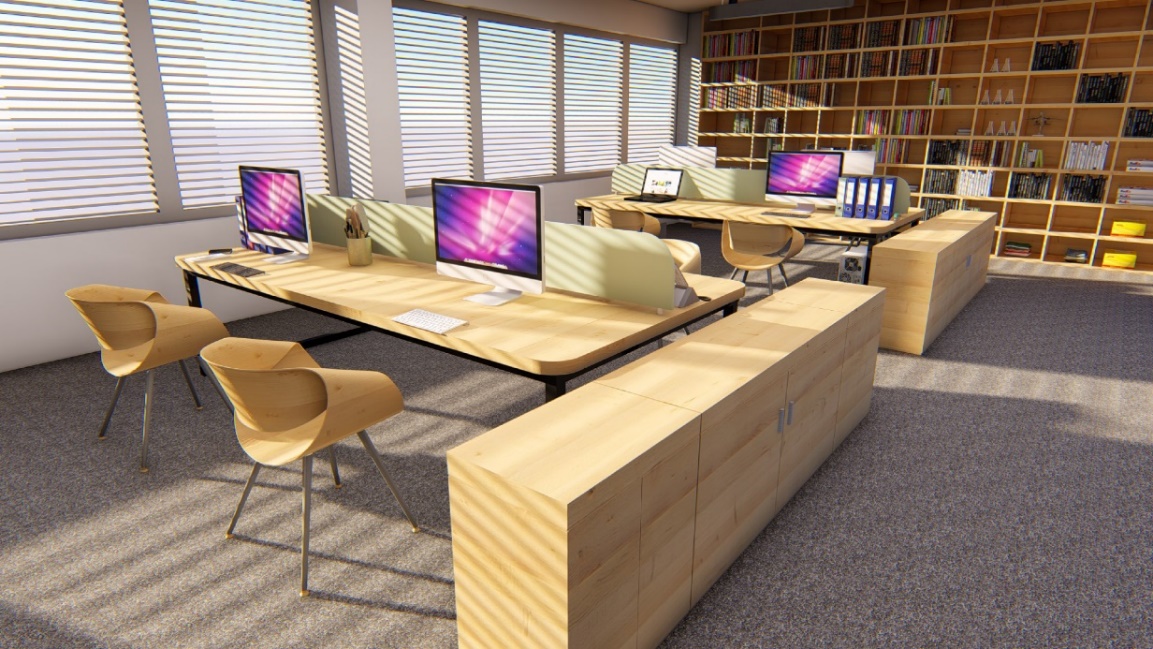


## Supplementary Figure 2. Light wood-colored and high-coverage wood

**
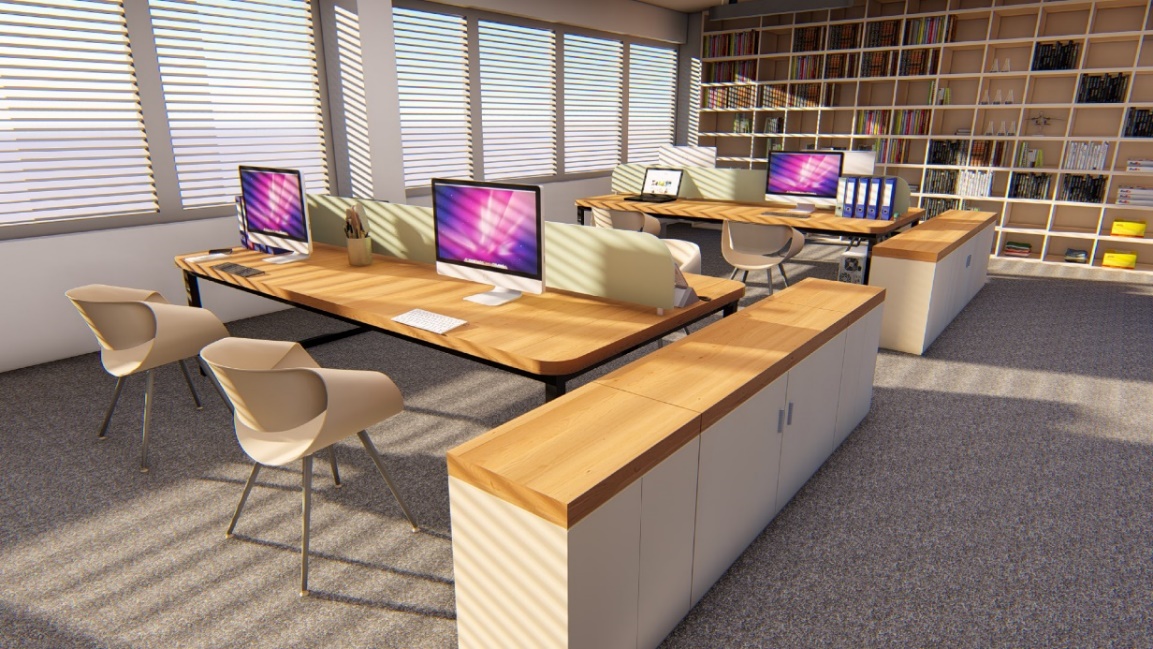
**

**Supplementary Figure 3.** Medium wood-colored and low-coverage wood

**
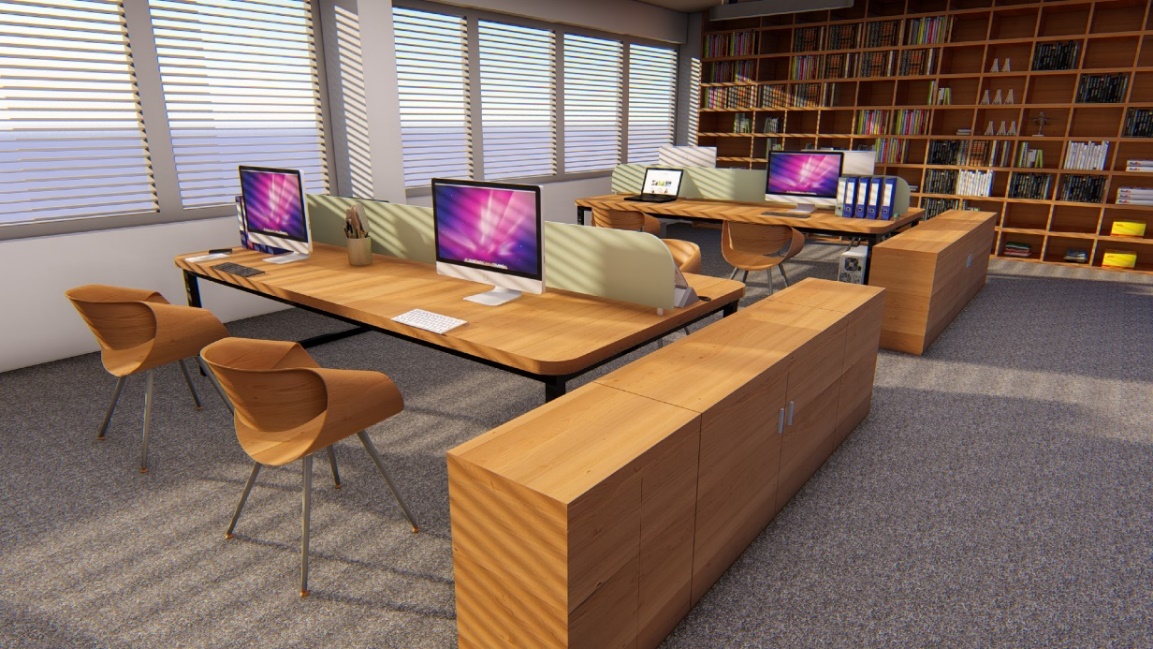
**

**Supplementary Figure 4.** Medium wood-colored and high-coverage wood


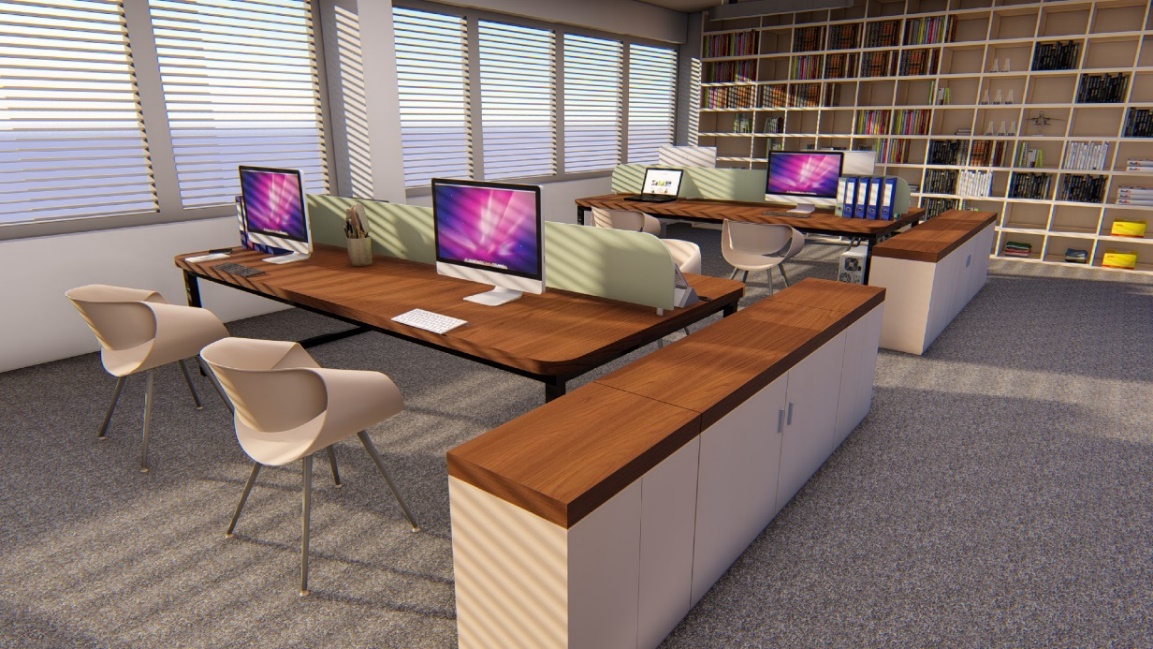


**Supplementary Figure 5.** Dark wood-colored and low-coverage wood


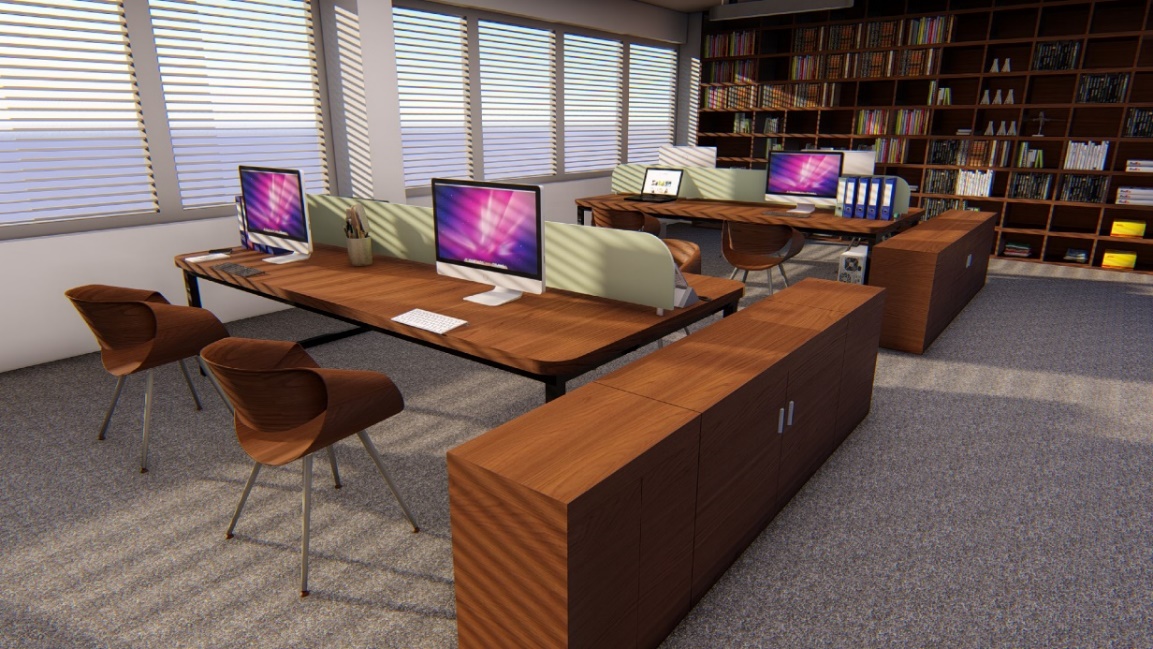


**Supplementary Figure 6.** Dark wood-colored and high-coverage wood


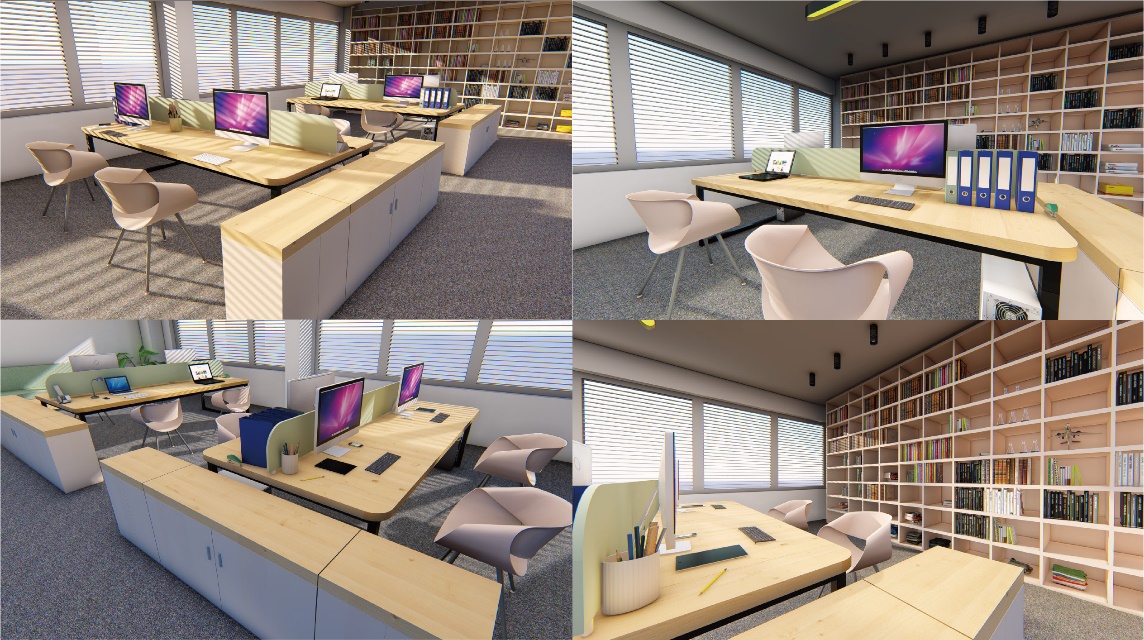


**Supplementary Figure 7.** Schematic diagram of the use scene of wooden office furniture (light & low)

**
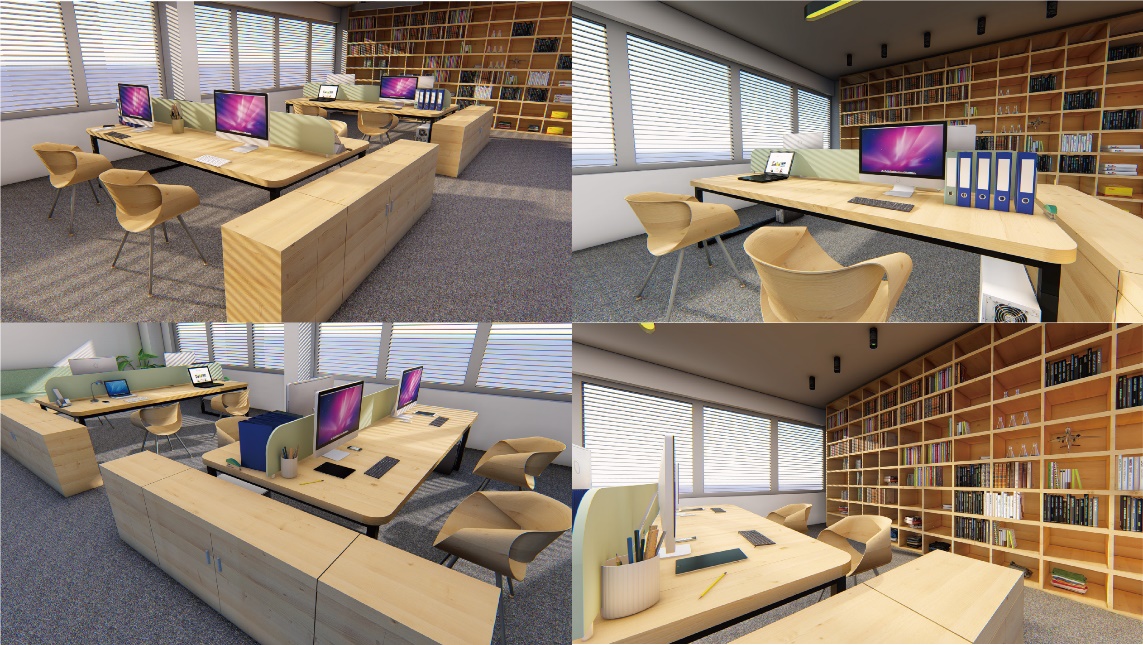
**

**Supplementary Figure 8.** Schematic diagram of the use scene of wooden office furniture(light & high)

**
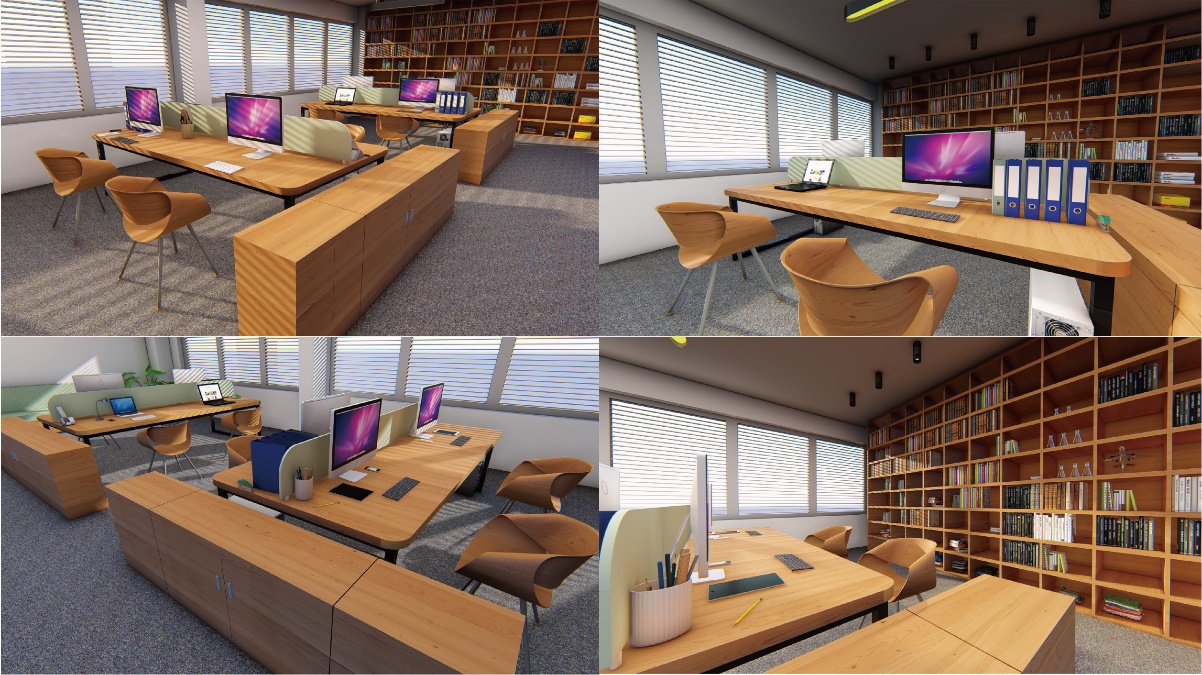
**

**Supplementary Figure 9.** Schematic diagram of the use scene of wooden office furniture(medium & high)

**
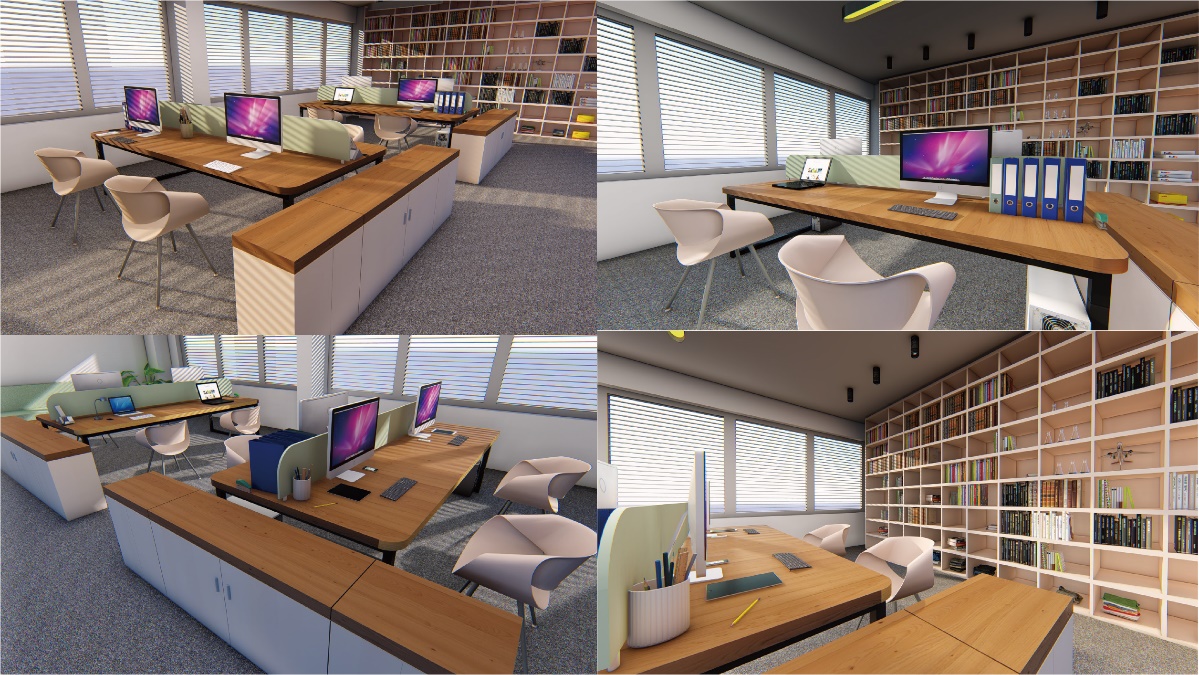
**

**Supplementary Figure 10.** Schematic diagram of the use scene of wooden office furniture(dark & low)

**
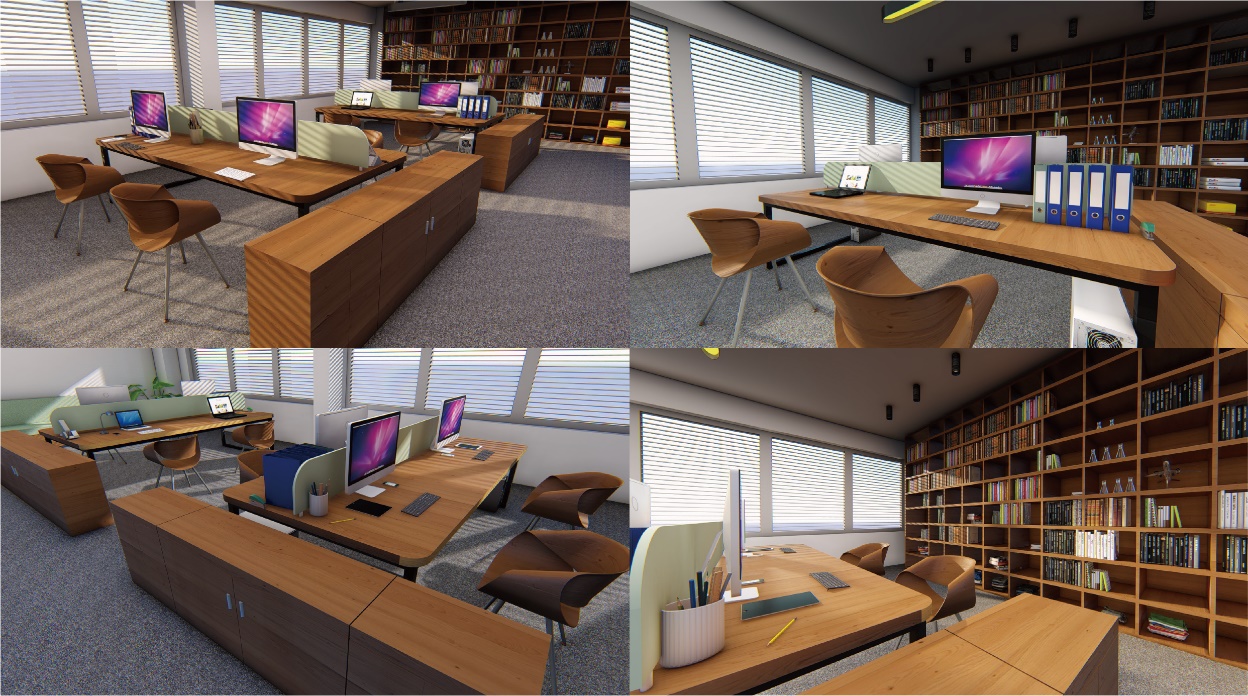
**

**Supplementary Figure 11.** Schematic diagram of the use scene of wooden office furniture(dark & high)
