# Supplementary figures and images for: Wood in office spaces: The impact of different wooden furniture on aesthetic evaluation
Source: Front Psychol. 2023 Jan 5;13:986627. doi: 10.3389/fpsyg.2022.986627 (PMC9849947; doi:10.3389/fpsyg.2022.986627)

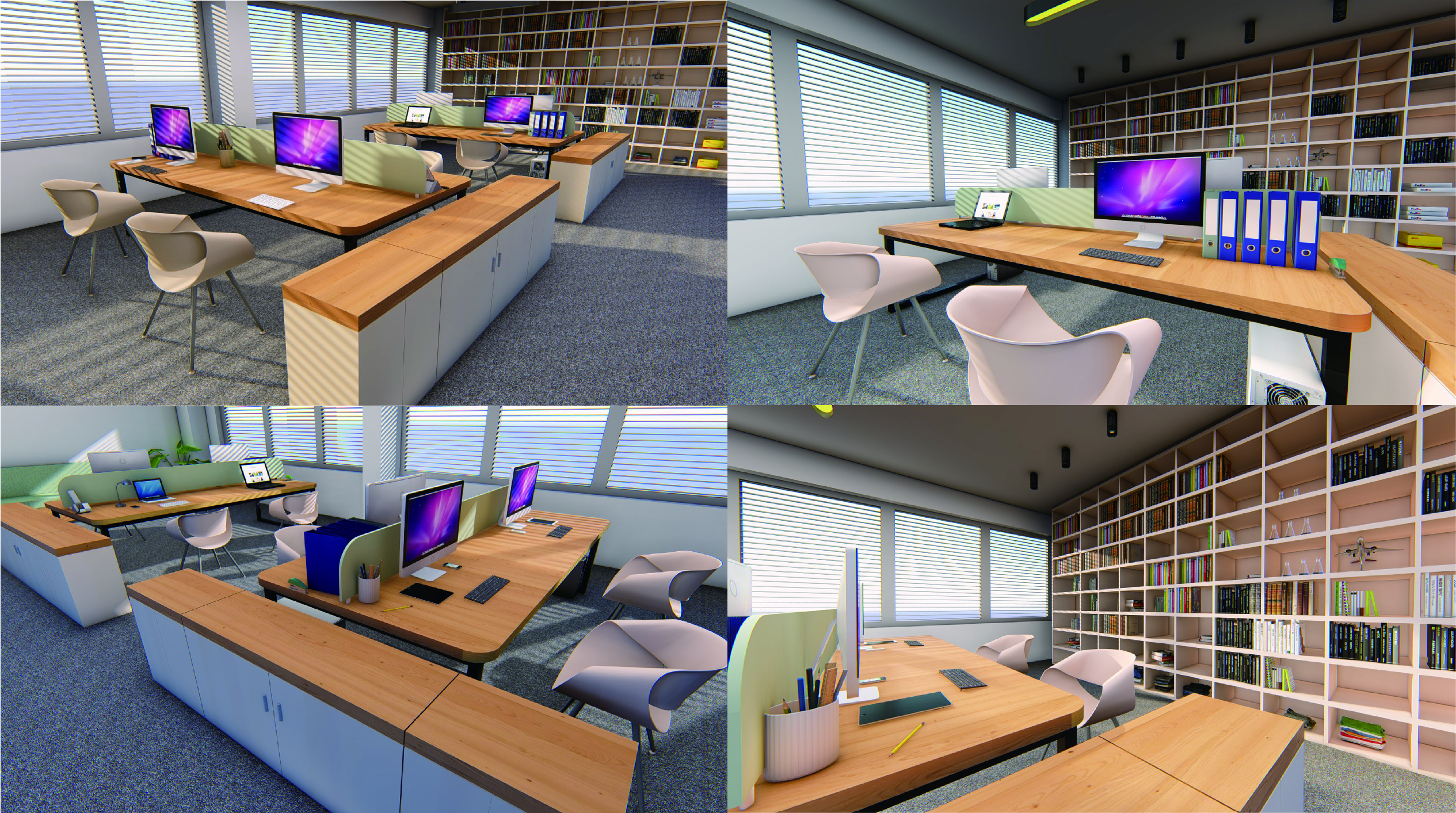

Supplement: Supplementary Image 1 — Schematic diagram of the use scene of wooden office furniture (medium and low). [file Image_1.jpg]
